# Ramadan Intermittent Fasting Affects Adipokines and Leptin/Adiponectin Ratio in Type 2 Diabetes Mellitus and Their First-Degree Relatives

**DOI:** 10.1155/2020/1281792

**Published:** 2020-07-28

**Authors:** Khaldoon Abdullah, Molham AL-Habori, Ekram Al-Eryani

**Affiliations:** Department of Biochemistry and Molecular Biology, Faculty of Medicine and Health Sciences, University of Sana'a, Sana'a, Yemen

## Abstract

**Background:**

In view of the association of Ramadan intermittent fasting with profound changes in lifestyle both in nondiabetic and diabetic patients, the aim of this study was to investigate the effect of Ramadan fasting on adiponectin, leptin and leptin to adiponectin ratio (LAR), growth hormone (GH), human-sensitive C-reactive protein (hs-CRP), and diabetic and metabolic syndrome factors in patients with Type 2 Diabetes Mellitus (Type 2 DM), their first-degree relatives (FDRs), and healthy controls.

**Methods:**

This cohort study involved 98 Yemeni male subjects aged 30-70 years old: 30 Type 2 DM, 37 FDRs of Type 2 diabetic patients, and 31 healthy control subjects. Subjects' body mass index (BMI), waist circumference (WC), and blood pressure (BP) were measured, and venous blood samples were collected twice: the first samples were collected a couple of days prior to Ramadan fasting (baseline) and the second samples after 3 weeks of fasting.

**Results:**

Ramadan fasting did not affect BMI, WC, and BP in Type 2 DM and their FDRs with respect to the baseline levels prior to Ramadan, whereas triglyceride and cholesterol were borderline significantly decreased in Type 2 DM with no effect in FDRs. Fasting blood glucose was not affected in Type 2 DM but was significantly increased in FDRs and control groups, whereas glycated haemoglobin (HbA1c) was slightly decreased in Type 2 DM, FDRs, and healthy controls. C-peptide, insulin, and insulin resistance (HOMA-IR) were significantly increased in Type 2 DM and FDRs, with no effect in the control group, whereas *β*-cell function (HOMA-*β*) was significantly decreased in FDRs and controls with no change in Type 2 DM. Ramadan fasting significantly decreased GH in both FDRs and control groups, and significantly increased hs-CRP in the control with no effect in Type 2 DM and FDRs. Adiponectin was significantly decreased, and leptin and LAR were significantly increased in Type 2 DM, FDRs, and control groups.

**Conclusion:**

Ramadan intermittent fasting decreased adiponectin and increased leptin, LAR, insulin, and insulin resistance in both Type 2 DM and FDRs as well as decreased GH in both FDRs and healthy controls and increased hs-CRP in healthy controls. Moreover, Ramadan intermittent fasting neither worsens a patient's glycemic parameters nor improves it, with the exception of a slight improvement in HbA1c in Type 2 DM, FDRs, and healthy controls.

## 1. Introduction

Ramadan is the holiest month in the Islamic calendar, in which Muslims fast during this month by refraining from all intakes of food, water, beverages, oral medicine, and smoking from sunrise till sunset [1]. The period of fasting may vary depending on the season of the year and the geographical location of the country. Therefore, daytime fasting may vary from approximately 11-18 hours, being longer in the summer [2]. During Ramadan, food and liquid are usually consumed in two meals, in the morning before sunrise and in the evening after sunset, shifting the pattern of energy intake from daytime to the hours of darkness [3, 4]. These changes in the timing of food intake as well as in the composition of diet can alter energy metabolism, and may affect important enzymatic, hormonal, and metabolic responses and different aspects of human health [4–9]. The type and quality of food eaten during the night in Ramadan may also be different from that usually consumed during the rest of the year [10].

Ramadan intermittent fasting is a safe lifestyle modification that involves specific dietary and lifestyle modifications, such as changes in food quantity and quality, nocturnal food consumption, meal frequency, and sleep cycle as well as reduction in exercise [11–14]. Ramadan fasting has been associated with variable weight changes, ranging from a modest weight gain to weight neutrality and weight loss [15], with a reported reduction in total calorie intake in some [16], but not all populations. Different studies have reported varying impacts of Ramadan intermittent fasting on overall health [2, 17]. Several studies though inconsistent have demonstrated the effects of Ramadan fasting on biochemical markers in healthy subjects [8, 9, 18–23]; subjects with obesity [24], metabolic syndrome (MetS) [12, 25], and hypertension [26, 27]; and in patients with cardiovascular disease [10, 28] and Type 2 diabetes mellitus [16, 29–32]. Such discrepancy in the results could be attributed to several confounding factors including age, gender, ethnicity, hours of fasting, number of fasting days, climatic conditions, cultural influences, sample size, study subjects, exercise, dietary patterns, and genetic background [1, 17, 33].

Despite the above-reported discrepancies between studies, Ramadan fasting remains an interesting alternative model to investigate the beneficial effect of intermittent fasting and its potential to mitigate chronic diseases in the general population [4, 34, 35]. There is, however, a general opinion that fasting is a potential nonpharmacological intervention for improving health and increasing longevity [17]. The beneficial effects of intermittent fasting on glycaemic control, metabolism, cardiovascular risk, cancer, and life expectancy have been researched [36, 37]. Energy and glucose metabolism, appetite, and hormonal responses are changed by prolonged fasting periods, and the changes in nutritional habits also affect lipid profile and body composition during Ramadan [13, 38]. For patients with Type 2 diabetes, Ramadan fasting implies major changes in their daily routines, including mealtimes, medication frequency and doses, daily activities, and sleeping quality. Consequently, there is an increased risk of affecting metabolic control in diabetic patients for a whole month annually [20, 39]. On the other hand, several studies reported that Ramadan fasting has a positive impact on Type 2 DM [31, 40] and that Ramadan fasting is unlikely to be hazardous for well-controlled patients.

In view of the association of Ramadan intermittent fasting with profound changes in lifestyle, such as altered sleeping durations and times and changes in physical activities as well as feeding patterns and restriction of food intake to night-time only, together with the existing controversial data in the literature on the effect of Ramadan fasting on metabolic and hormonal factors, this study is aimed at investigating the effect of Ramadan fasting on adipocytokines (adiponectin, leptin, and leptin/adiponectin ratio (LAR)), growth hormone (GH), human-sensitive C-reactive protein (hs-CRP) (as a marker of systemic inflammation), diabetic parameters, and metabolic syndrome factors in Type 2 DM and their first-degree relatives (FDRs) and healthy controls.

## 2. Methods

### 2.1. Subjects and Data Collection

This cohort study involved 98 Yemeni male subjects aged 30-70 years old: 30 Type 2 DM patients recruited during their routine visits to the Endocrine and Diabetic Clinic of 48 Model Hospital, Sana'a, with fasting blood glucose > 126 mg/dl and had been diagnosed with diabetes for at least one year and on oral hypoglycaemic drugs and desired to fast during Ramadan; 37 healthy first-degree relatives of the subjects with Type 2 DM who accompanied their diabetic patients; and 31 healthy control subjects with fasting blood glucose (FBG) < 100 mg/dl, who were on no medication that may affect blood glucose or lipid profile. Subjects with acute or chronic illness, or secondary diabetes mellitus due to other conditions or under immunosuppression (malignancy, renal failure, liver cirrhosis, connective tissue disease, and chronic congestive heart failure) were excluded from the study. The study was carried out in the period of June 2016 (Ramadan of 2016), and the study protocol was approved by the Institutional Review Board (IRB) of the Faculty of Medicine and Health Sciences, Sana'a University.

All participants were recruited from 48 Model Hospital, Sana'a, via posters. Volunteers were approached 2 weeks before Ramadan and were given information sheets explaining the research procedure, specimen collection methods, and a written informed consent. All participants were asked to read and sign the consent form prior to the commencement of the study. Each participant was then assessed at two visits: a couple of days prior to Ramadan fasting (baseline) and a follow-up visit after 3 weeks of fasting. Baseline demographical and clinical data including age, duration of diabetes, family history of diabetes, diet and lifestyle, the current treatment regimen that the patient is following, history of hypertension, dyslipidaemia, and diabetes mellitus complications were collected from each participant. All participants were subjected to physical measurements, and fasting blood samples were obtained for laboratory analysis.

The subjects' height and weight were measured and body mass index (BMI), defined as weight (kg)/height squared (m^2^), was calculated. Waist circumference (WC) was measured halfway between the lower rib margin and the anterior superior iliac spine. Blood pressure (BP) measurements were taken from each patient's right arm in the seated position by using an Omron IntelliSense Automatic Blood Pressure Monitor after 10 min of rest. Two to three successive BP readings were obtained at 5-minute intervals and averaged. Venous blood (5 ml) was collected from each individual after an overnight fast of more than 10 hours and divided into two vacuumed tubes; 4 ml was put into plain tubes for biochemical assay, and 1 ml was put into a K2EDTA tube for glycated haemoglobin (HbA1c) determination. The serum from each sample was separated within 30 minutes and aliquoted into four Eppendorfs and immediately kept at -80°C for biochemical analysis. Haemolysates were prepared immediately for HbA1c determination within 2 hours of blood collection.

### 2.2. Biochemical Analysis

Fasting blood glucose (FBG), triglyceride (TG), total cholesterol, HDL-cholesterol (HDL-c), and LDL-cholesterol (LDL-c) were measured on an automated analyzer, the Cobas c501 (Roche Diagnostic, Germany), using the respective Roche Diagnostic kits (Mannheim, Germany). Glycated haemoglobin (HbA1c) was measured on the Cobas c501 automated analyzer (Roche Diagnostic, Germany) using a turbidimetric inhibition immunoassay. Insulin, C-peptide, and GH were measured by an electrochemiluminescence immunoassay (ECL) on an Elecsys autoanalyzer (Roche Diagnostic, Germany). Insulin resistance (HOMA-IR), insulin sensitivity (HOMA-S), and *β*-cell function (HOMA-*β*) were calculated using the Homeostasis Model Assessment (HOMA2) Calculator v2.2 which is available from the Oxford Centre for Diabetes, Endocrinology, and Metabolism [41]. Serum hs-CRP was measured by the immunoturbidimetric method (Roche Diagnostic, Germany) on the Cobas c501 automated analyzer (Roche Diagnostic, Germany). Enzyme-linked immunoassay (ELISA) kits were used to measure serum adiponectin and leptin (Société de Pharmacologie et d'Immunologie BIO, France).

### 2.3. Statistical Analysis

The results were analyzed by the Statistical Package for the Social Sciences (SPSS) version 20 (SPSS Inc., Chicago, IL, USA), and the results were expressed as mean ± SD with the exception of hs-CRP (not normally distributed) which is reported as geometric means ± 95%confidence intervals, following its log transformation. The significance and mean differences of all parameters among the three tested groups were assessed by ANOVA, whereas the significance and mean differences of all parameters prior to and during Ramadan was assessed by a paired *t*-test. The significant interrelationships between parameters were analyzed by Pearson's correlation, with the exception of hs-CRP which was analyzed by Spearman's correlation. Significant differences were indicated if *p* value was <0.05.

## 3. Results

Table 1 shows the comparison of demographic, biochemical, and hormonal parameters between control, FDRs, and Type 2 DM subjects prior to Ramadan. In FDRs, both BMI and SBP were significantly (*p* = 0.028, *p* = 4.9 × 10^−7^) higher by 11.6% and 7.1% as compared to the control group, whereas WC and DBP were nonsignificantly different. Triglyceride was nonsignificantly higher and total cholesterol was significantly (*p* = 1.1 × 10^−8^) higher in FDRs by 7.2% and 19.5% as compared to the control group, with no significant effect on both HDL-c and LDL-c. Fasting blood glucose, HbA1c, and C-peptide were nonsignificantly higher in FDRs, whereas insulin was borderline significantly (*p* = 0.054) higher by 31.8% and both HOMA-IR and HOMA-*β* were nonsignificantly higher by 30.3% and 16.6% than that of the control group. However, HOMA-S was significantly (*p* = 0.003) lower in FDRs by 25.4% with respect to the control group. Adiponectin and GH were nonsignificantly lower in FDRs by 6% and 6.3% as compared to the control group, whereas both leptin and leptin/adiponectin ratio (LAR) were nonsignificantly higher in FDRs by 18.3% and 27% and hs-CRP was borderline significantly (*p* = 0.055) higher by 35.1%.

In Type 2, DM, age, BMI, WC, and SBP were significantly (*p* = 0.004,*p* = 5.1 × 10^−9^;*p* = 0.004, *p* = 5.1 × 10^−9^) higher in Type 2 DM by 45%, 15.2%, 9.9%, and 11.8%, respectively, as compared to the control group, with no significant effect on DBP. Triglyceride and total cholesterol were significantly (*p* = 0.014; *p* = 2.7 × 10^−8^) higher in Type 2 DM by 30.2% and 19.5% as compared to the control group, with no significant effect on both HDL-c and LDL-c. Fasting blood glucose, HbA1c, and C-peptide were significantly (*p* = 5.1 × 10^−9^; *p* = 5.1 × 10^−9^; and *p* = 5.2 × 10^−9^) higher in Type 2 DM with respect to the control group by 70.7%, 59.2%, and 94.8%, respectively. Similarly, insulin and HOMA-IR were significantly (*p* = 5.2 × 10^−9^) higher in Type 2 DM (by 2.3-fold and 2.5-fold) than in the control group. In contrast, HOMA-*β* was borderline significantly (*p* = 0.06) lower in Type 2 DM by 20.3% as compared to the control group, and HOMA-S was significantly (*p* = 1 × 10^−6^) lower by 62.4%. Adiponectin and GH were significantly (*p* = 0.001; *p* = 5.1 × 10^−9^) lower in Type 2 DM by 17.7% and 58.9% with respect to the control group, whereas leptin, leptin/adiponectin ratio (LAR), and hs-CRP were significantly (*p* = 5.1 × 10^−9^; *p* = 2.8 × 10^−8^; *p* = 0.002) higher by 4-fold, 4.8-fold, and 54.1%, respectively.

However, on comparing Type 2 DM with FDRs, FBG, HbA1c, and C-peptide were significantly (*p* = 5.1 × 10^−9^; *p* = 5.1 × 10^−9^; and *p* = 1.4 × 10^−7^) higher in Type 2 DM as compared to FDRs by 66.6%, 49.9%, and 58.5%, respectively. Similarly, insulin and HOMA-IR were also significantly (*p* = 5.2 × 10^−9^; *p* = 5.1 × 10^−9^) higher in Type 2 DM with respect to FDRs by 73.3% and 91%, respectively. In contrast, both HOMA-*β* and HOMA-S were significantly (*p* = 1 × 10^−4^; *p* = 9 × 10^−6^) lower in Type 2 DM by 31.7% and 49.6% with respect to FDRs. Adiponectin and GH were significantly (*p* = 0.038; *p* = 5.1 × 10^−9^) lower in Type 2 DM by 12.5% and 56.1% with respect to FDRs, whereas leptin and LAR were significantly (*p* = 5.15 × 10^−9^; *p* = 1.0 × 10^−6^) higher in Type 2 DM by 3.3-fold and 3.8-fold, respectively, and hs-CRP was nonsignificantly higher in Type 2 DM by 14%.

Table 2 shows the effect of Ramadan fasting on the biochemical and hormonal parameters among control, FDRs and Type 2 DM. In the control group, TG was significantly (*p* = 0.038) decreased by 5.7% and LDL-c was significantly (*p* = 1.7 × 10^−8^) increased by 7.7% with respect to baseline prior to Ramadan, with no effect on both total cholesterol and HDL-c. On the other hand, FBG was significantly (*p* = 4.1 × 10^−11^) increased by 17%, with no significant effect on C-peptide, insulin, and HOMA-IR. In contrast HbA1c and HOMA-*β* were significantly (*p* = 0.041; *p* = 2.8 × 10^−8^) decreased by 3.9% and 26.3%, respectively, as compared to the baseline. However, HOMA-S was borderline (*p* = 0.059) significantly decreased by 6.6%. On the other hand, GH was significantly (*p* = 2.4 × 10^−8^) decreased by 18.2%, and hs-CRP was significantly (*p* = 0.009) increased by 29.7% as compared to the baseline. Adiponectin was significantly (*p* = 3.3 × 10^−7^) decreased by 7%, whereas leptin and LAR were significantly (*p* = 3.4 × 10^−8^; *p* = 7.2 × 10^−9^) increased by 24.4% and 33.3% with respect to the baseline prior to Ramadan.

In FDRs, metabolic syndrome factors (TG, total cholesterol, HDL-c, and LDL-c) were not significantly affected by Ramadan fasting. In contrast, diabetic parameters such as FBG, C-peptide, insulin, and HOMA-IR were significantly (*p* = 2.2 × 10^−13^; *p* = 3 × 10^−6^; 0.001; and *p* = 4.8 × 10^−5^) increased by 11.8%, 13.6%, 13%, and 16%, respectively, as compared with the baseline prior to Ramadan. On the other hand, HbA1c, HOMA-*β*, and HOMA-S were significantly (*p* = 1.1 × 10^−14^; *p* = 0.01; and *p* = 0.005) decreased by 9.6%, 11.7%, and 15%, respectively, with respect to the baseline. Growth hormone was significantly (*p* = 1.2 × 10^−9^) decreased by 25%, whereas hs-CRP was not affected as compared to the baseline. Adiponectin was significantly (*p* = 2.6 × 10^−7^) decreased by 6.7%, whereas leptin and LAR were significantly (*p* = 5 × 10^−5^; *p* = 1.3 × 10^−5^) increased by 15.23% and 24.1% with respect to the baseline prior to Ramadan.

In Type 2 DM, of the metabolic syndrome factors, TG and total cholesterol were borderline significantly (*p* = 0.06; *p* = 0.08) decreased by 6% and 5.1% with respect to the baseline prior to Ramadan fasting, with no effect on HDL-c and LDL-c. However, diabetic parameters such as C-peptide, insulin, and HOMA-IR were significantly (*p* = 1 × 10^−6^; *p* = 1.4 × 10^−9^; *p* = 1.3 × 10^−7^) increased by 14.4%, 16.3%, and 15.4%, respectively, as compared to the baseline, with no significant effect on FBG. In contrast, HbA1c and HOMA-S were significantly (*p* = 1 × 10^−6^; *p* = 3 × 10^−6^) decreased by 10.4% and 14.2%, with no significant effect on HOMA-*β*. On the other hand, GH was nonsignificantly decreased by 5.1% and hs-CRP was nonsignificantly affected with respect to the baseline prior to Ramadan fasting. Adiponectin was significantly (*p* = 0.005) decreased by 7.4%, whereas leptin and LAR were significantly (*p* = 9 × 10^−5^; *p* = 0.022) increased by 4.1% and 15.1%, respectively, as compared to the baseline prior to Ramadan.

Table 3 shows the significant interrelationships between adiponectin, leptin, hs-CRP, and GH with the diabetic and metabolic syndrome factors in control, FDRs, and Type 2 DM groups during Ramadan as assessed by Pearson's correlation analysis. In the control group, adiponectin was negatively correlated with BMI, WC, leptin, and LAR (*r* = −0.90, *p* < 0.0001; *r* = −0.59, *p* = 0.020; *r* = −0.90, *p* < 0.0001; and *r* = −0.92, *p* < 0.0001; respectively), and that of leptin was positively correlated with BMI, WC, and LAR (*r* = 0.90, *p* < 0.0001; *r* = 0.76, *p* < 0.001; *r* = 0.99, *p* < 0.0001; respectively). Leptin was also observed to be weakly positively associated with insulin and HOMA-IR (*r* = 0.53, *p* < 0.043; *r* = 0.53, *p* < 0.044). However, in FDRs, adiponectin was negatively correlated with BMI, WC, TG, leptin, and LAR (*r* = −0.91, *p* < 0.0001; *r* = −0.83, *p* < 0.0001; *r* = −0.69, *p* = 0.004; *r* = −0.86, *p* < 0.0001; and *r* = −0.93, *p* < 0.0001; respectively). Leptin was positively correlated with BMI, WC, and LAR (*r* = 0.87, *p* < 0.0001; *r* = 0.73, *p* = 0.002; and *r* = 0.98, *p* < 0.0001; respectively). However, hs-CRP was weakly positively correlated with BMI, insulin, and HOMA-IR (*r* = 0.33, *p* = 0.046; *r* = 0.37, *p* = 0.023; *r* = 0.37, *p* = 0.024). Along the same line, in Type 2 DM, adiponectin was negatively correlated with BMI, WC, leptin, and LAR (*r* = −0.95, *p* < 0.0001; *r* = −0.52, *p* = 0.045; *r* = −0.75, *p* = 0.001; and *r* = −0.85, *p* < 0.0001; respectively), and leptin was positively correlated with BMI, insulin, HOMA-IR, and LAR (*r* = 0.85, *p* < 0.0001; *r* = 0.62, *p* = 0.014; *r* = 0.63, *p* = 0.012; and *r* = 0.96, *p* < 0.0001; respectively). Growth hormone was negatively correlated with FBG, insulin, and HOMA-IR (*r* = −0.56, *p* = 0.001; *r* = −0.79, *p* < 0.0001; *r* = −0.79, *p* < 0.0001; respectively). However, hs-CRP was weakly positively correlated with WC (*r* = 0.43, *p* = 0.019).

## 4. Discussion

Although there have been reports on metabolic changes during and after Ramadan in healthy subjects and in patients with diabetes, the results are conflicting [22, 42–44]. The results presented in this study demonstrate that the association of Ramadan intermittent fasting with profound changes in lifestyle, such as altered sleeping durations and times, and changes in physical activities as well as in feeding patterns and restriction of food intake to night-time only decreased adiponectin and increased leptin, LAR, insulin, and insulin resistance in both Type 2 DM and their FDRs as well as decreased GH in both FDRs and healthy controls and increased hs-CRP in healthy controls.

In our study, adiponectin was significantly decreased by Ramadan fasting in Type 2 DM, FDRs, and healthy control groups, which is in agreement with studies reporting a significant decrease in adiponectin level during the fasting period in healthy controls [22, 45] and in overweight/obese individuals [46], and a decrease in the expression of adiponectin in patients with Type 2 DM [47]. Other studies, however, reported that adiponectin either increased in healthy controls that have risk factors for Type 2 DM [48, 49] or did not change in healthy individuals [7, 42]. The observed decrease in adiponectin can be explained by the altered sleeping/feeding pattern during Ramadan, as well as by the previously reported changes in cortisol secretion [9], where the adiponectin concentrations exhibit ultradian pulsatility, as well as diurnal variation, with a significant nocturnal decline, reaching minimum values in the early morning [50]. In a similar manner to shift workers, individuals fasting on the month of Ramadan experience a severe disturbance in their sleeping patterns, with loss of night-time sleep and shortening of sleep duration. This can be associated with the loss of the circadian rhythm of cortisol, a hormone that controls the expression of many other hormones and cytokines including adipokines [9]. This loss of circadian rhythmicity might be related to alterations in the expression of CLOCK genes, resulting in hypercortisolism and chronic inflammation, increasing the risk of chronic cardiometabolic disorders [33, 51]. Profound changes in the diurnal expression of CLOCK, a central component of the circadian molecular clock, were demonstrated during Ramadan compared to the nonfasting month of Shabaan [33]. Moreover, sleep deprivation over time during Ramadan leads to elevation of inducing proinflammatory cytokines, such as IL-6 which will decrease the level of adiponectin and exert insulin resistance [51].

On the other hand, leptin and LAR were significantly increased by Ramadan fasting in Type 2 DM, FDRs, and healthy control groups, which might also be attributed to the changes in sleeping patterns, with a complete reversal of the sleep vs. wake cycle, whereby this sleep disturbance/sleeping quality is considered as a stressor leading to hypercortisolism. It may also result from the change in feeding pattern to night-time with an increase in total caloric intake during nonfasting hours (night) supported by significant insulin elevation during Ramadan fasting with respect to baseline prior to Ramadan. Both altered cortisol secretion and sleeping/feeding pattern will cause an increase in leptin after the meal (at night) and a decrease in adiponectin (ultradian pulsatility) leading to a significantly increased insulin resistance [9]. Our results are consistent with previous studies reporting increased levels of leptin during Ramadan fasting in healthy controls [42, 52] and in overweight/obese individuals [46]. Other studies, on the other hand, found either no significant change in leptin levels during Ramadan fasting in Type 2 DM, obese, and healthy controls [53–55] or a decreased leptin level during Ramadan fasting [22, 56]. Moreover, the observed decrease of adiponectin and increased leptin and LAR in Type 2 DM, their FDRs, and the healthy controls during Ramadan could also be attributed to adiposity, whereby adiponectin was inversely correlated and leptin was positively correlated with BMI and WC in Type 2 DM, FDRs, and healthy controls, which are in line with a number of studies [57, 58]. This negative correlation between adiponectin and WC may be due to the fact that adiponectin gene expression is downregulated in obesity [57]. Previously, we reported that adiponectin and leptin were associated more with obesity and less with diabetes [59].

Analogous to the pattern observed prior to Ramadan, the GH levels in Type 2 DM and FDRs were significantly lower than those of the control group during Ramadan, which is in agreement with other studies [44] and may be attributed to the elevated insulin and cortisol levels during the diurnal day of Ramadan fasting [9]. On examining the effect of Ramadan fasting, GH was also significantly decreased in both FDRs and healthy control groups, which is in agreement with an earlier finding [22]. This may most likely be due to differences in sleeping and meal patterns, and it is likely to contribute to insulin resistance in FDRs, reflecting the strong association between GH and that of hyperglycemia and hyperinsulinemia [60]. It is well known that GH increased with exercise [61]. Hence, the less exercise exerted by participants during Ramadan fasting could also contribute to the observed decrease in GH levels. However, an earlier study concluded that long-term fasting in Ramadan could not affect GH significantly, whereas short-term fasting could [44].

In contrast, Ramadan fasting significantly increased hs-CRP in healthy controls with no effect in both Type 2 DM and FDRs. The increased hs-CRP in healthy controls may be attributed to the sleep disturbance during this month, whereby hs-CRP has been reported to be elevated immediately after sleep restriction and sustained for two days after recovery of sleep [62]. Several studies, however, have shown that intermittent Ramadan fasting enhances the inflammatory state in healthy individuals by the reduction of inflammatory markers such as hs-CRP [9, 33, 63, 64] and attributed the downregulation of inflammatory cytokine to the reduced body fat percentage and caloric restriction during the month of Ramadan [65]. Liver protein synthesis was also reported to be more significantly decreased by fasting compared to other organs [66]. In addition, consumption of the daily food all in one meal distorted the circadian rhythm, particularly when it was taken in the morning, whereby a morning meal increases the total 24-hour synthesis of protein in liver, whereas an evening meal did not [22]. On the other hand, the lack of effect in our Type 2 DM and their FDRs may offer an opportunity to reduce low-grade systemic inflammation and oxidative stress, as well as the subsequent adverse health effects in these groups, considering the significantly higher hs-CRP in Type 2 DM prior to Ramadan with respect to the control group as well as the observed positive correlation of hs-CRP with insulin resistance in FDRs prior to Ramadan implicating chronic inflammation in the development of insulin resistance and Type 2 DM.

Ramadan fasting was observed to significantly increase C-peptide, insulin, and HOMA-IR in Type 2 DM and FDRs with respect to the baseline prior to Ramadan, with no effect in the healthy controls, which are in line with those observed in obese individuals characterized by increased insulin resistance [67–69]. This slight but significant increase of insulin and insulin resistance by Ramadan fasting may be attributed to the reverse feeding schedule which causes metabolism disruption with increased caloric intake and sleep time alteration where sleep was reported to have important modulatory effects on glucose regulation, and recurrent sleep loss was associated with marked negative alterations of the parameters of glucose tolerance [70]. This reverse feeding schedule and sleep time alteration together with decreased physical activity during Ramadan results in modulatory hormonal changes due to complex interactions mediated by GH secretion during the first part of the night (in response to muscle relaxation and sleeping onset) [61] and by cortisol secretion during the second part of the night due to changes in cortisol secretory patterns (diurnal rhythm of plasma cortisol) leading to decreased glucose tolerance and increased insulin resistance [22]. Interestingly, however, insulin sensitivity (HOMA-S) was significantly decreased in both Type 2 DM and FDRs, with an observed significant decrease in *β*-cell function (HOMA-*β*) in FDRs and no change in Type 2 DM. On the other hand, the observed lack of effect in our healthy control group is not in accordance with studies reporting increased insulin resistance in healthy and obese individuals [9, 67–69]; however, it was in line with other studies reporting that Ramadan fasting enhanced insulin resistance [45, 53] and with other studies reporting that Ramadan fasting had no effect/change in trained young men as compared to pre-Ramadan [7]. This lack of effect is further supported by the significant decrease of HOMA-*β* and the borderline significant decrease in HOMA-S in the healthy control group.

Moreover, our study indicates that Ramadan fasting neither worsens a patient's glycemic parameters nor improves it, with the exception of a slight improvement in HbA1c in Type 2 DM, FDRs, and healthy controls. The latter is in agreement with previous studies showing decreased HbA1c in Type 2 DM, FDRs, and healthy individuals [16, 67, 71–73]. However, a single study showed that HbA1c was not remarkably affected by Ramadan fasting [53]. On the other hand, FBG was not affected by fasting during Ramadan in our Type 2 diabetics, which is in agreement with several studies [2, 53, 74]. A recent study also suggested that except for an initial increase in glucose variability, fasting during Ramadan for patients with non-insulin-treated Type 2 DM did not cause any significant changes in metabolic control, glucose fluctuation, or time in hypoglycaemia during a continuous glucose monitoring and recording period compared to the nonfasting pre-Ramadan period [75]. Other studies, however, reported that Ramadan fasting increased FBG in Type 2 DM [76] and attributed it to a defect in the balance between circulating levels of insulin and counterregulatory hormones during prolonged fasting as well as to overeating during nonfasting hours of Ramadan and change in dosage of antidiabetic drugs to prevent hypoglycemia in individuals with Type 2 DM [77, 78]. Unlike the observed effect on Type 2 DM, our results showed that FBG was significantly increased (within normal range) in both FDRs and healthy controls with respect to their baseline levels prior to Ramadan, which is in agreement with several studies [10, 68, 79]. This observed increase could be attributed to a major change in dietary patterns (Ramadan nutrition habits) like consumption of substantial quantities of sugary fluids (juice and carbonated drinks) together with fried foods and carbohydrate rich meals after breaking the fast and during the night or may be due to insulin resistance in FDRs.

The results presented in this study are in line with previous reports showing that Ramadan fasting has a modest effect on anthropometric, carbohydrate, and lipid metabolism in Type 2 DM and healthy individuals [1, 67, 80]. Despite the marked changes in food habits during Ramadan, our study showed no significant effect on BMI of Type 2 DM and their FDRs with respect to the baseline levels prior to Ramadan, which is in agreement with studies showing no difference in BMI in Type 2 DM, healthy individuals, and obese males [4, 11, 24, 80–82]. This was also confirmed by the EPIDIAR study reporting unchanged body weight in the majority of Type 2 DM [39]. Studies examining the effect of Ramadan fasting on variations in eating behavior, appetite ratings, satiety efficiency, and energy expenditure in healthy participants reported no significant differences in anthropometric measures before Ramadan compared to during Ramadan and after Ramadan, and no significant difference in resting metabolic rate and total energy expenditure before and after Ramadan [13, 83]. Other studies, however, did show a decrease in weight during Ramadan [10, 16, 21, 23, 69, 84]. However, a recent study suggested that reported weight changes with Ramadan in other studies are more likely to be due to differences in food intake and might be suggestive of a shift towards fat rather than carbohydrate as source of fuel during Ramadan [13]

Moreover, consistent with previous studies, our results also showed no difference in waist circumference [16] and blood pressure [30] in Type 2 DM. However, an earlier study [53, 73] showed that blood pressure decreased during Ramadan as compared to before Ramadan, whereas a more recent study concluded that Ramadan fasting increased blood pressure in Type 2 diabetic patients [84]. On the other hand, a significant decrease was reported in the waist circumference of patients with Type 2 DM [84], metabolic syndrome [12], and in healthy controls [69] during Ramadan. In addition, our results also showed a borderline significant decrease in TG and cholesterol in Type 2 DM with no effect in FDRs. However, a significant decrease in TG and a significant increase in LDL-c were observed in the healthy control group. The decrease in TG is in line with those reported in several studies [10, 85] and in disagreement with other studies reporting an increase in TG [69, 86]. These changes in blood lipids, however, seem to be variable and depend probably on the quality and quantity of food consumption and the degree of weight changes [2]. A recent study concluded that Ramadan fasting may not affect body composition and characteristics of metabolic syndrome markers in healthy adult men [80].

The strength of our study is the assessment of Ramadan intermittent fasting in 3 groups: healthy controls, a group with a high risk for developing Type 2 DM (FDRs), and Type 2 diabetic patients. However, the limitations of our study include the small sample size of the three tested groups and the lack of details concerning the type of food consumed, the physical activity, and sleep duration of the participants. All the parameters tested were evaluated only prior to and at the end of the Ramadan fasting; thus, it is uncertain whether the changes observed persisted over an extended period after Ramadan fasting. The patients who participated in our study were not matched for age and BMI and were controlled diabetics and thus are not representative of all patients with Type 2 DM.

## 5. Conclusion

This study showed that Ramadan fasting significantly decreased adiponectin and significantly increased leptin and LAR in Type 2 DM, FDRs, and control groups, as well as significantly decreased GH in FDRs and healthy controls and significantly increased hs-CRP in the control group. These changes may most likely be due to alterations in sleeping (with complete reversal of sleep vs. wake cycle) and meal patterns (consumption of the daily food all in one meal) and caloric restriction with increased total caloric intake during nonfasting hours (night) as well as reduced physical activity exerted during Ramadan fasting. Moreover, Ramadan fasting showed slight improvement in HbA1c level in Type 2 DM, FDRs, and healthy controls as compared to those levels prior to Ramadan, with no noticeable effect on body composition and overall characteristics of metabolic syndrome markers. Over all it seems that the possibly intended benefits of Ramadan fasting/intermittent fasting may be offset by the dysregulation in the diurnal rhythm during Ramadan. Therefore, to maximize the health benefits of intermittent fasting, it is highly recommended to control the present day sleep disturbance and unnecessary caloric intake during Ramadan.

## Figures and Tables

**Table 1:** Biochemical and hormonal levels in healthy controls, FDRs, and Type 2 DM prior to Ramadan fasting.

Parameters	Control (*n* = 31)	FDRs (*n* = 37)	Type 2 DM (*n* = 30)	*P* value		
				C vs. F	C vs. D	F vs. D
Age (years)	34.61 ± 4.31	34.35 ± 3.83	50.17 ± 12.95	0.990	5.1 × 10^−9^	5.1 × 10^−9^
BMI (kg/m^2^)	22.18 ± 3.05	24.76 ± 4.37	25.55 ± 4.49	0.028	0.004	0.701
WC (cm)	90.86 ± 8.53	92.94 ± 9.03	99.83 ± 14.20	0.708	0.004	0.028
SBP (mmHg)	111.16 ± 3.33	119.00 ± 5.34	124.30 ± 4.11	4.9 × 10^−7^	5.1 × 10^−9^	0.004
DBP (mmHg)	81.74 ± 3.37	81.78 ± 2.69	83.30 ± 2.49	0.999	0.274	0.403
Triglyceride (mg/dl)	118.10 ± 36.00	126.65 ± 50.41	153.79 ± 57.40	0.752	0.014	0.066
Cholesterol (mg/dl)	154.75 ± 19.58	184.98 ± 29.01	184.69 ± 35.31	1.1 × 10^−8^	2.74 × 10^−8^	0.999
HDL-c (mg/dl)	39.57 ± 7.12	37.25 ± 7.32	38.30 ± 9.55	0.465	0.810	0.858
LDL-c (mg/dl)	103.62 ± 25.00	112.27 ± 23.69	111.15 ± 28.12	0.349	0.485	0.983
FBG (mmol/l)	4.47 ± 0.35	4.58 ± 0.49	7.63 ± 2.23	0.937	5.1 × 10^−9^	5.1 × 10^−9^
HbA1c (%)	5.19 ± 0.36	5.51 ± 0.48	8.26 ± 1.75	0.404	5.1 × 10^−9^	5.1 × 10^−9^
C-peptide (ng/ml)	0.96 ± 0.42	1.18 ± 0.44	1.87 ± 0.53	0.120	5.2 × 10^−9^	1.4 × 10^−7^
Insulin (pmol/l)	99.37 ± 40.62	130.98 ± 46.43	226.95 ± 75.05	0.054	5.1 × 10^−9^	5.2 × 10^−9^
HOMA-IR	1.78 ± 0.71	2.32 ± 0.82	4.43 ± 1.55	0.095	5.1 × 10^−9^	5.1 × 10^−9^
HOMA-*β* (%)	178.52 ± 49.84	208.19 ± 58.08	142.27 ± 76.59	0.127	0.064	1 × 10^−4^
HOMA-S (%)	66.32 ± 26.43	49.51 ± 20.99	24.96 ± 7.77	0.003	1 × 10^−6^	9 × 10^−6^
GH (ng/ml)	1.92 ± 0.69	1.80 ± 0.44	0.79 ± 0.20	0.565	5.1 × 10^−9^	5.1 × 10^−9^
hs-CRP^∗^ (mg/l)	0.37 (0.31-0.42)	0.50 (0.40-0.59)	0.57 (0.49-0.65)	0.055	0.002	0.360
Leptin (ng/ml)	20.03 ± 8.70	23.70 ± 7.91	79.22 ± 26.33	0.819	5.1 × 10^−9^	5.15 × 10^−9^
Adiponectin (IU/ml)	19.04 ± 2.18	17.90 ± 2.66	15.67 ± 2.28	0.397	0.001	0.038
LAR	1.11 ± 0.61	1.41 ± 0.69	5.29 ± 2.29	0.883	2.8 × 10^−8^	1 × 10^−6^

Data are presented as means ± SD. ^∗^Data are presented as geometric mean and 95% confidence interval of mean evaluated by ANOVA. C vs. F: a comparison between control and FDRs; C vs. D: a comparison between control and Type 2 DM; F vs. D: a comparison between FDRs and Type 2 DM.

**Table 2:** Effect of Ramadan fasting on biochemical and hormonal levels in healthy controls, FDRs, and Type 2 DM.

Groups	Control			FDRs			Type 2 DM		
	Baseline	Ramadan	*P* value	Baseline	Ramadan	*P* value	Baseline	Ramadan	*P* value
BMI (kg/m^2^)	22.18 ± 3.05	22.14 ± 3.04	0.070	24.76 ± 4.37	24.70 ± 4.31	0.108	25.55 ± 4.49	25.51 ± 4.38	0.293
WC (cm)	90.86 ± 8.53	90.75 ± 8.59	0.085	92.94 ± 9.03	92.91 ± 8.89	0.629	99.83 ± 14.20	99.61 ± 14.27	**0.024**
SBP (mmHg)	111.16 ± 3.33	111.38 ± 3.51	0.622	119.00 ± 5.34	118.28 ± 6.47	0.547	124.30 ± 4.11	124.00 ± 6.16	0.811
DBP (mmHg)	81.74 ± 3.37	82.41 ± 3.64	0.392	81.78 ± 2.69	81.78 ± 3.26	1.00	83.30 ± 2.49	84.30 ± 2.59	0.090
TG (mg/dl)	118.10 ± 36.00	111.33 ± 32.89	**0.038**	126.65 ± 50.41	124.64 ± 50.75	0.642	153.79 ± 57.40	144.56 ± 51.37	0.060
Cholesterol (mg/dl)	154.75 ± 19.58	159.93 ± 26.25	0.198	184.98 ± 29.01	178.38 ± 29.57	0.133	184.69 ± 35.31	175.27 ± 33.73	0.081
HDL-c (mg/dl)	39.57 ± 7.12	40.24 ± 6.23	0.549	38.48 ± 6.75	38.48 ± 6.75	0.259	38.30 ± 9.55	37.34 ± 7.11	0.395
LDL-c (mg/dl)	103.62 ± 25.00	111.57 ± 23.91	1.7 × 10^−8^	112.27 ± 23.69	112.18 ± 26.36	0.978	111.15 ± 28.12	106.03 ± 30.15	0.371
FBG (mmol/l)	4.47 ± 0.35	5.23 ± 0.42	4.1 × 10^−11^	4.58 ± 0.49	5.12 ± 0.56	2.2 × 10^−13^	7.63 ± 2.23	7.47 ± 1.80	0.651
HbA1c (%)	5.19 ± 0.36	4.99 ± 0.60	**0.041**	5.51 ± 0.48	4.98 ± 0.44	1.1 × 10^−14^	8.26 ± 1.75	7.40 ± 1.49	1 × 10^−6^
C-peptide (ng/ml)	0.96 ± 0.42	0.90 ± 0.40	0.079	1.18 ± 0.44	1.34 ± 0.50	3 × 10^−6^	1.87 ± 0.53	2.14 ± 0.61	1 × 10^−6^
Insulin (pmol/l)	99.37 ± 40.62	99.69 ± 40.25	0.919	130.98 ± 46.48	147.96 ± 55.48	**0.001**	226.95 ± 75.05	263.90 ± 86.25	1.4 × 10^−9^
HOMA-IR	1.78 ± 0.71	1.85 ± 0.73	0.155	2.32 ± 0.82	2.69 ± 1.01	4.8 × 10^−5^	4.43 ± 1.55	5.11 ± 1.74	1.3 × 10^−7^
HOMA-*β* (%)	178.52 ± 49.84	131.65 ± 37.07	2.8 × 10^−8^	208.19 ± 58.08	183.76 ± 59.11	**0.010**	142.27 ± 76.59	149.08 ± 50.34	0.663
HOMA-S (%)	66.23 ± 26.43	61.84 ± 21.79	0.059	49.51 ± 20.99	42.11 ± 18.00	**0.005**	24.96 ± 7.77	21.41 ± 5.89	3 × 10^−6^
GH (ng/ml)	1.92 ± 0.69	1.57 ± 0.46	2.4 × 10^−8^	1.80 ± 0.44	1.35 ± 0.27	1.2 × 10^−9^	0.79 ± 0.20	0.75 ± 0.21	0.230
hs-CRP^∗^ (mg/l)	0.37 (0.31-0.42)	0.48 (0.41-0.54)	**0.009**	0.50 (0.40-0.59)	0.51 (0.42-0.59)	0.744	0.57 (0.49-0.65)	0.58 (0.42-0.59)	0.871
Leptin (ng/ml)	20.03 ± 8.70	24.91 ± 7.79	3.4 × 10^−8^	23.70 ± 7.91	27.37 ± 9.15	5 × 10^−5^	79.22 ± 26.33	82.46 ± 26.23	9 × 10^−5^
Adiponectin (IU/ml)	19.04 ± 2.18	17.70 ± 2.33	3.3 × 10^−7^	17.90 ± 2.66	16.70 ± 2.60	2.6 × 10^−7^	15.67 ± 2.28	14.51 ± 2.51	**0.005**
LAR	1.11 ± 0.61	1.48 ± 0.67	7.2 × 10^−9^	1.41 ± 0.69	1.75 ± 0.84	1.3 × 10^−5^	5.29 ± 2.29	6.09 ± 3.00	**0.022**

Data are presented as means ± SD. ^∗^Data are presented as geometric mean and 95% confidence interval of mean evaluated by ANOVA. Significant difference were indicated when *p* < 0.05 (which are highlighted in bold).

**Table 3:** Pearson's correlation coefficient of adiponectin, leptin, hs-CRP, and GH with diabetic and metabolic syndrome factors in control, FDRs, and Type 2 DM groups during Ramadan.

	Adiponectin	Leptin	hs-CRP	GH
Control				
BMI	-0.899 (<0.0001)	0.897 (<0.0001)	0.155 (0.406)	-0.010 (0.967)
WC	-0.589 (0.021)	0.755 (0.001)	0.250 (0.175)	0.276 (0.133)
Insulin	-0.422 (0.117)	0.529 (0.043)	-0.266 (0.174)	-0.055 (0.767)
HOMA-IR	-0.327 (0.235)	0.526 (0.044)	-0.251 (0.174)	-0.056 (0.763)
GH	-0.260 (0.349)	0.410 (0.129)	0.316 (0.084)	1
hs-CRP	0.052 (0.853)	-0.123 (0.662)	1	0.316 (0.084)
Leptin	-0.896 (<0.0001)	1	-0.123 (0.662)	0.410 (0.129)
Adiponectin	1	-0.896 (<0.0001)	0.052 (0.853)	-0.260 (0.349)
LAR	-0.924 (<0.0001)	0.995 (<0.0001)	-0.111 (0.694)	0.384 (0.158)
First-degree relatives				
BMI	-0.912 (<0.0001)	0.867 (<0.0001)	0.331 (0.046)	0.231 (0.170)
WC	-0.833 (0.0001)	-0.728 (0.002)	0.318 (0.055)	0.337 (0.042)
TG	-0.694 (0.004)	0.513 (0.051)	0.279 (0.094)	0.232 (0.168)
Insulin	-0.355 (0.194)	0.180 (0.521)	0.373 (0.023)	0.022 (0.897)
HOMA-IR	-0.378 (0.165)	0.208 (0.456)	0.371 (0.024)	0.036 (0.832)
GH	-0.561 (0.029)	0.701 (0.004)	-0.048 (0.776)	1
hs-CRP	-0.243 (0.383)	-0.037 (0.897)	1	-0.048 (0.776)
Leptin	-0.856 (<0.0001)	1	-0.037 (0.897)	0.701 (0.004)
Adiponectin	1	-0.859 (<0.0001)	0.243 (0.383)	-0.561 (0.029)
LAR	-0.928 (<0.0001)	0.978 (<0.0001)	0.078 (0.175)	0.689 (0.005)
Type 2 diabetes				
BMI	-0.950 (<0.0001)	0.851 (<0.0001)	0.292 (0.117)	-0.358 (0.052)
WC	-0.524 (0.045)	0.460 (0.085)	0.426 (0.019)	-0.162 (0.394)
FBG	-0.348 (0.789)	0.495 (0.061)	-0.114 (0.548)	-0.558 (0.001)
Insulin	-0.458 (0.086)	0.621 (0.014)	0.124 (0.514)	-0.786 (<0.0001)
HOMA-IR	-0.460 (0.084)	0.628 (0.012)	0.095 (0.618)	-0.793 (<0.0001)
GH	0.371 (0.174)	-0.657 (0.008)	-0.257 (0.170)	1
hs-CRP	-0.255 (0.359)	0.056 (0.844)	1	-0.257 (0.170)
Leptin	-0.751 (0.001)	1	0.056 (0.844)	-0.657 (0.008)
Adiponectin	1	-0.751 (0.001)	-0.255 (0.359)	0.371 (0.174)
LAR	-0.851 (<0.0001)	0.957 (<0.0001)	0.119 (0.674)	-0.631 (0.012)

## Data Availability

The dataset generated and/or analyzed during this study is included in this submitted manuscript and is available from the corresponding author on reasonable request.

## Abbreviations

MetS: Metabolic syndrome
LAR: Leptin/adiponectin ratio
GH: Growth hormone
hs-CRP: Human-sensitive C-reactive protein
FDRs: First-degree relatives
FBG: Fasting blood glucose
BMI: Body mass index
WC: Waste circumference
BP: Blood pressure
HbA1c: Glycated haemoglobin
TG: Triglycerides
HDL-c: High-density lipoprotein cholesterol
LDL-c: Low-density lipoprotein cholesterol
ECL: Electrochemiluminescence immunoassay
HOMA: Homeostasis model assessment
HOMA-IR: Insulin resistance
HOMA-S: Insulin sensitivity
HOMA-*β*: *β*-Cell function
ELISA: Enzyme-linked immunoassay
SD: Standard deviation
SPSS: Statistical Package for Social Sciences
SBP: Systolic blood pressure
DBP: Diastolic blood pressure.
